# Synthesis of N‐Doped Graphene Photo‐Catalyst for Photo‐Assisted Charging of Li‐Ion Oxygen Battery

**DOI:** 10.1002/gch2.202300166

**Published:** 2023-12-07

**Authors:** Nilay Kaçar, Ersu Lökçü, Meltem Çayirli, Reşat Can Özden, Sahin Coskun, Cigdem Toparli, İbrahim Çelikyürek, Mustafa Anik

**Affiliations:** ^1^ Department of Metallurgical and Materials Engineering Eskisehir Osmangazi University Eskisehir 26040 Turkey; ^2^ Department of Metallurgical and Materials Engineering Middle East Technical University Ankara 06800 Turkey; ^3^ Present address: Department of Metallurgical and Materials Engineering Bursa Technical University 16310 Bursa Turkey

**Keywords:** chemical vapor deposition, Li‐Ion oxygen batteries, N‐doped graphene, photo‐catalyst, photo‐charging

## Abstract

In this work, nitrogen (N)‐doped graphene film is synthesized, as a photo‐catalyst, on one side of the copper foam by chemical vapor deposition and the copper foam is directly used as an electrode after porous Pd@rGO cathode loading to the other side of the foam for the photo‐assisted charging of the Li‐ion oxygen battery. The amount of urea (CO(NH_2_)_2_), which is used as N atom source, is optimized to get maximum photo‐anodic currents from the n‐type graphene films. The optical band gap and the valance band edge potential of the optimized N‐doped graphene film are determined as 2.00 eV and 3.71 V_Li+/Li_, respectively. X‐ray photoelectron spectra provided that the atomic percent of N atoms in the graphene film is 1.34% and the graphitic, pyrrolic and pyridinic N atom percentages are 54.01%, 42.20% and 3.79%, respectively. The photo‐assisted charging tests indicated that the N‐doped graphene film photo‐catalyst reduced the charging potential significantly even at 1000 mA g^−1^ (0.1 mA cm^−2^) current density and improved the cyclic discharge‐charge performance of the Li‐ion oxygen battery considerably.

## Graphical abstract

Nitrogen (N)‐doped graphene film is synthesized as a photo‐catalyst for the photo‐assisted charging of the Li‐ion oxygen battery. After structural and the optical characterizations, the effectiveness of the photo catalyst is determined by the charge/discharge tests that N‐doped graphene film photo‐catalyst reduced the charging potential and improved the cyclic performance of the Li‐ion oxygen battery considerably.

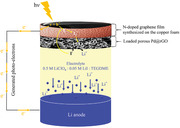

## 1 Introduction

The photo‐assisted charging with the aid of triiodide/iodide (I_3_^−^/I^−^) redox shuttling was reported as a very effective approach to reduce the extended charging overpotential, which results from the sluggish oxidation kinetics of the low conducting Li_2_O_2_, for the Li‐ion oxygen batteries.^[^ ^1^, ^2^ ^]^ During the illumination of the n‐type semiconductor photo‐catalyst, the created holes are consumed in the oxidation of I^−^ ions to I_3_^−^ ions, which are reduced back to I^−^ ions on the oxygen electrode, and the photo‐excited electrons transferred to the anode are used in the reduction of lithium cations.^[^ ^1^ ^]^ Thus the generated photo‐voltage, which is mainly determined by the difference between the Li^+^/Li redox potential and photo‐electrode conduction band (CB) edge potential, contributes to the reduction of the charging potential.^[^ ^1^ **^]^**

Graphene is a very well‐known semimetal with extraordinary electrical and optical properties.^[^ ^3^, ^4^ ^]^ Tailoring of its electronic structure (and thus tuning of the Fermi level position), however, is needed through controllable doping for many applications.^[^ ^5^, ^6^, ^7^, ^8^ ^]^ Nitrogen (N) atom doping into the graphene structure is very common to get the n‐type semiconductor since N has one more valance electron than carbon (C). The substitution of C atoms by N in a very stable 2D honeycomb lattice necessitates the doping process to be carried out during the synthesis of graphene at high temperatures. Therefore, chemical vapor deposition (CVD) is a very versatile and widely used technique in the synthesis and doping of graphene.^[^ ^9^, ^10^ ^]^ The N atom is found as pyridinic N, pyrrolic N, and graphitic N in the graphene lattice depending on the bonding structure.^[^ ^11^ ^]^ Nitrogen atom formed covalent bonds with two nearby C atoms in a six‐membered ring is referred as pyridinic N. If these covalent bonds form with two nearby C atoms in a five‐membered ring then the N atom is referred as pyrrolic N. Nitrogen atoms simply replaced by C atoms in the six‐membered ring are called graphitic N.

Plenty of semiconductor materials in various forms were synthesized as photo‐catalyst in order to use them in the photo‐assisted charging of the Li‐ion oxygen batteries. Dye‐sensitized TiO_2_,^[^ ^1^ ^]^ g‐C_3_N_4_,^[^ ^2^ ^]^ TiN/TiO_2_/carbon cloth,^[^ ^12^ ^]^ Au/TiO_2_ nanotubes,^[^ ^13^ ^]^ TiO_2_/Fe_2_O_3_,^[^ ^14^ ^]^ WO_3_/g‐C_3_N_4_^[^ ^15^ ^]^ and rGO/g‐C_3_N_4_^[^ ^16^ ^]^ can be given as examples. All these semiconductors are synthesized in one or multi‐step processes and then loaded onto the electrode to be used in the cell. Separately synthesizing, transferring and loading sequences of the active materials always suffer a variety of complications like oxidation, hydration or loading with inhomogeneous thickness. The thickness control of the photo‐catalyst material is very crucial to get an efficient amount of photo‐excited electrons without allowing electron‐hole recombination. In this work, 2D N‐doped graphene was synthesized on one side of the Cu foam by CVD technique and the other side of the Cu foam was loaded by the porous Pd@rGO (cathode; oxygen electrode). Then the Cu foam was directly used as an electrode (in fact as a current collector) in the Li‐ion oxygen battery cell. This novel procedure eliminated the separate bulk photo‐catalyst loading steps and the photo‐catalyst with only a few atomic layers obtained homogeneously on the electrode surface to ensure a significant reduction in the electron‐hole recombination. Efficient generation of the photo‐excited electrons with the 2D photo‐catalyst resulted in the charging of the Li‐ion oxygen battery at a very high current density without reaching an extreme charging potential in our work.

## 2 Experimental Section

### 2.1 Synthesis Methods

The synthesis details of the palladium‐loaded porous reduced graphene oxide (Pd@rGO nanostructures), which was used as a cathode (oxygen electrode), can be found in the previous work.^[^ ^16^ ^]^

**Figure 1:**
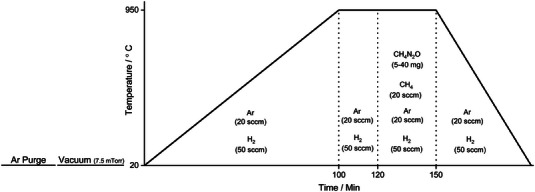
The synthesis procedure of the N‐doped graphene films by CVD.

The N‐doped graphene was synthesized by CVD according to the procedure provided in **Figure** **1**. For the characterizations and the electrochemical tests like photo‐current and Mott‐Schottky measurements, copper foil substrate (Sigma‐Aldrich, 25 µm thick, 99.999% purity) was used in the synthesis of the N‐doped graphene. After cleaning, 1 cm × 1 cm copper foil substrate was placed in the hot zone of CVD. Urea powder (CO(NH_2_)_2_; Sigma‐Aldrich, ACS, >99.5% purity) was used as the N atom source and it was placed in the cold zone of CVD. During the growth of the graphene, urea powder was drawn into the zone at 60 °C–75 °C and by the end of the growth process it was assured that all the urea powder was evaporated. After the synthesis process N‐doped graphene film on the copper foil was coated with poly methyl methacrylate (PMMA) on the spin coater and then the copper substrate was dissolved in 50% HCl solution including 0.5 M FeCl_3_. PMMA was also cleaned by the acetone/isopropanol combinations after the coated graphene film was transferred to either SiO_2_/Si or tem grit for the characterizations, or to ITO‐coated glass (Rs < 50 ohm ^−1^sq) for the electrochemical measurements. The copper foam (MTI Corporation, >99.9% purity and 550 g m^−^2) was used instead of copper foil as the substrate in the synthesis of the N‐doped graphene in order to use it directly as an electrode in the photo‐assisted discharge/charge tests of the Li‐ion oxygen battery.

### 2.2 Electrode Preparations and Electrochemical Measurements

The photo‐current and Mott‐Schottky measurements were conducted in a conventional three‐electrode cell (N‐doped graphene films transferred to the surface of ITO‐coated glass as the working electrode, a platinum wire as the auxiliary electrode and an Ag/AgCl (saturated KCl) as the reference electrode) with a Gamry Reference 3000 workstation. A solar simulator (A‐type 150 W, 1–3 SUN, Xenon lamb, AMO filters; 400–700 nm wavelength) was used as the light source and tests were conducted in a spectral cell containing 0.1 m KCl buffered by 0.1 m K_2_HPO_4_ to pH 7.

The pictures of the homemade oxygen cabin and a cell, which were used in the photo‐assisted charging tests, were provided previously.^[^ ^16^ ^]^ All the test assemblages were carried out in an Ar‐filled glove box with H_2_O and O_2_ levels less than 0.1 ppm. Lithium metal was used as both counter and reference electrodes and the glass microfiber filter (Whatman) was used as a separator. Porous Pd@rGO/Super P carbon black/PVDF were mixed (80:10:10 wt.%) in NMP and the slurry was coated onto the back side of the copper foam (the front side contained N‐doped graphene film) with a loading rate of 0.1 mg cm^−2^ as a cathode. The capacity of the Li‐ion oxygen battery was calculated according to the geometrical area of the cathode surface since only 9% of the 2D surface area was calculated as holes after cathode active material loading on Cu foam (Figure S1, Supporting Information). The cathode side of the foam was facing the anode and the photo‐electrode side of the foam was facing the spectral glass window mounted on the homemade cell. 0.5 M LiClO_4_ and 0.05 M LiI dissolved in TEGDME in order to use as an electrolyte in the designed cell. The galvanostatic discharge and charge test cut‐off potentials were 2.0 V_Li+/Li_ and 3.6 V_Li+/Li_, respectively. The current densities were changing between 10 mA g^−1^ (10^−3^ mA cm^−2^) and 1000 mA g^−1^ (10^−1^ mA cm^−2^).

### 2.3 Structural Characterizations

The graphene film morphology was examined with a ZEISS Ultraplus scanning electron microscope (SEM). X‐ray photoelectron spectroscopy (XPS, PHI 5000 Versa Probe spectrometer) with Al Kα radiation was performed. Standard C 1s spectrum at 284.6 eV was used for the calibration of the peaks. The PHI 5000 charge compensation system was utilized to prevent localized charge accumulation. The graphene film morphology was also characterized using a JEOL JEM 2010F TEM running at 200 kV. Atomic force microscopy (AFM, Veeco Innova) measurement was performed to investigate surface morphology. UV‐vis spectra were obtained by Cary 5000 UV‐vis‐NIR spectrometer with diffuse reflectance accessory between 200 and 800 nm. The Raman analysis was carried out by Raman spectroscopy (RENISHAW RAMAN in Via Microscope) with an excitation laser of 633 nm.

## 3 Results and Discussion

### 3.1 Structural and Optical Properties of N‐Doped Graphene

**Figure 2:**
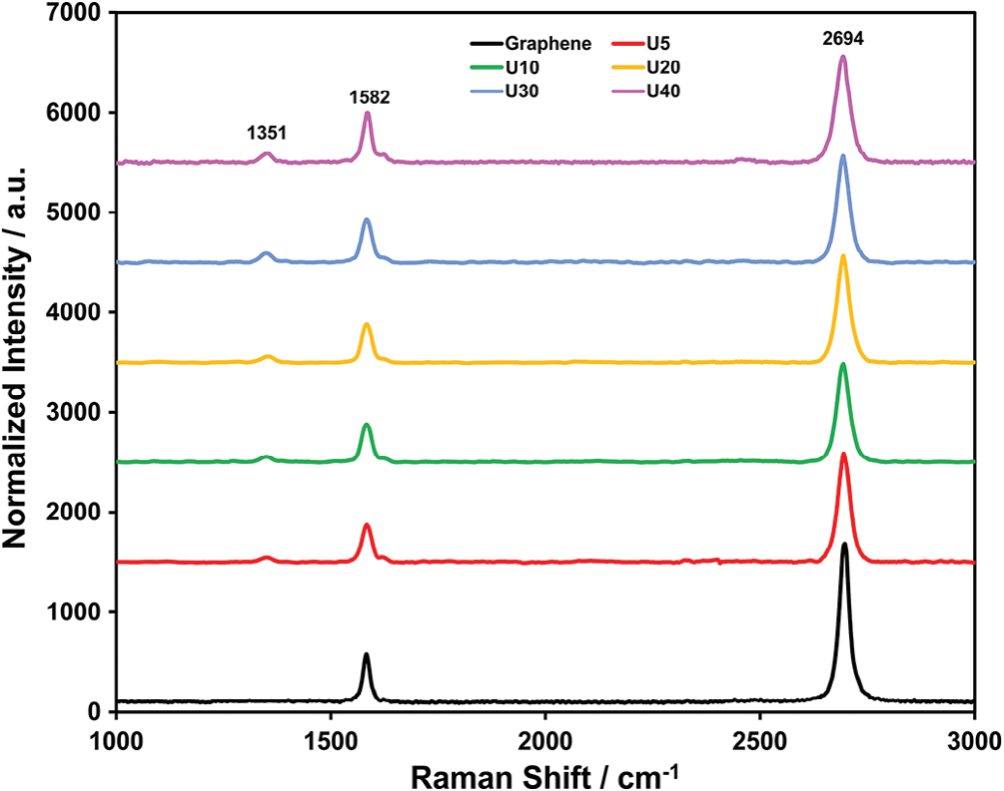
The normalized Raman spectra obtained from the pure graphene and N‐doped graphene films.

5 mg, 10 mg, 20 mg, 30 mg, and 40 mg urea (CO(NH_2_)_2_) per cm^2^ of copper substrate were used as the N‐dopant for the graphene films which were designated as U5, U10, U20, U30, and U40, respectively. The normalized Raman spectra from the graphene films are provided in **Figure** **2**. Three main peaks can be assigned in the Raman Spectrum. The D band (≈1351 cm^−1^), which is absent in the pure graphene film, indicates the atomic doping that insertions of N atoms in the sp^2^ C matrices result in topological defects^[^ ^17^ ^]^ in the N‐doped films. As the amount of urea used in the CVD process increases, the intensity of the D band increases slightly for the N‐doped graphene films in Figure 2. The G (≈1582 cm^−1^) and 2D (≈2694 cm^−1^) bands are attributed to the sp^2^ hybridizations of the C atoms.^[^ ^18^ ^]^ The 2D band is sensitive to the number of layers of the graphene films.^[^ ^18^ ^]^ The 2D/G intensity ratio is 3.3 for the pure graphene film and it varies from 2.9 to 2.5 for the N‐doped graphene films, which look to have a few layers.

**Figure 3:**
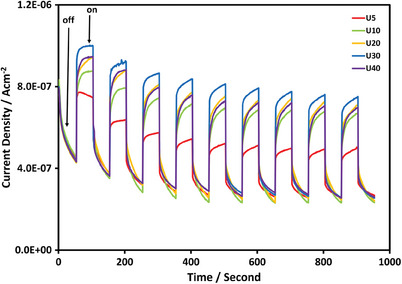
The photo‐anodic currents of N‐doped graphene films obtained at 1 V_Ag/AgCl_ via on‐off cycles after transferring to the ITO‐coated glass surface.

The photo‐anodic currents, via on‐off cycles under the visible‐light irradiation, were determined for all the synthesized N‐doped graphene films at 1 V_Ag/AgCl_ as in **Figure** **3**. The photo‐anodic currents show a sharp increase upon increase in urea from 5 mg cm^−2^ to 10 mg cm^−2^ and then small incremental increases are seen in the photo‐anodic currents as the urea increases up to 30 mg cm^−2^ Probably 30 mg cm^−2^ urea provides a kind of saturation doping level in the graphene films at our experimental conditions. Above 30 mg cm^−2^ urea, however, the photo‐anodic currents start to decrease probably because of the deterioration in the film structure since the higher amount of urea corrodes the Cu foil substrate at the CVD temperature. Therefore, 30 mg cm^−2^ urea is used as the N source in the synthesis of the N‐doped graphene films in this study.

The SEM, TEM and AFM images of the synthesized U30 films are given in Figure S2 (Supporting Information). SEM image (Figure S2a (Supporting Information): on TEM grit) shows that the substrate is covered with a continuous graphene film during the synthesis. Figure S2b (Supporting Information) presents a typical TEM image of the few‐layer graphene films. The scattered diffraction spots in the ring‐shaped pattern indicate that the N atom insertions disturb the crystalline structure of graphene. The continuous multi‐layer graphene film can also be observed in the AFM image in Figure S2c (Supporting Information) that the N doping process by the usage of urea as the N atom source does not cause the graphene film to break into the small islands.

**Figure 4:**
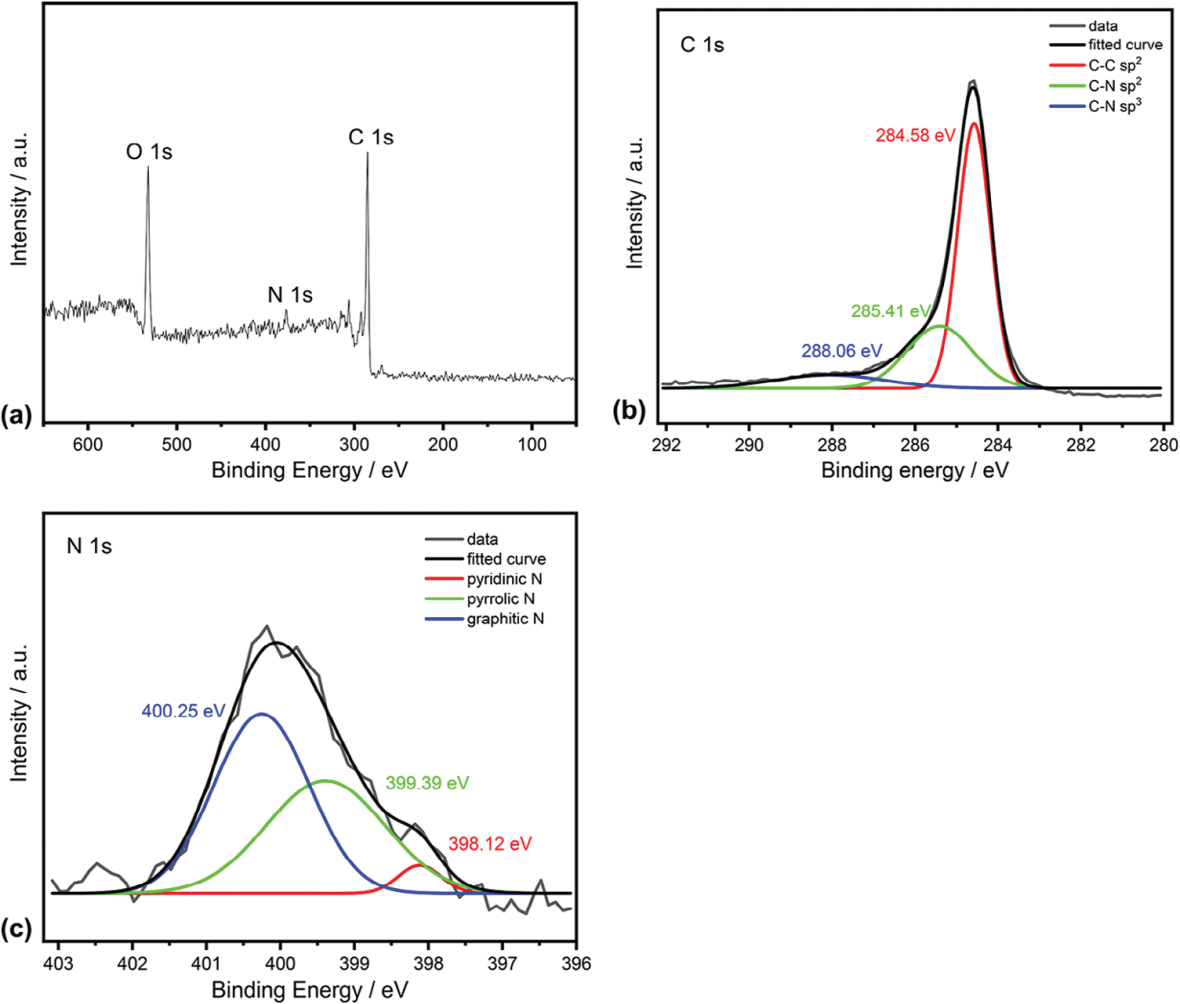
a) The XPS spectra b) C 1s spectrum and c) N 1s spectrum of the N‐doped graphene (U30).

The doping process in the graphene film can be better understood by getting the XPS spectra to determine the configuration and concentration of the N atoms in the graphene structure. The XPS spectra, C 1s spectrum and N 1s spectrum of the N‐doped graphene (U30) are provided in **Figure** **4**a–c, respectively. In the XPS spectra (Figure 4a), the peaks at 285.00, 396.10, and 532.10 eV correspond to C 1s of sp^2^ C, N 1s of the doped N, and O 1s of the absorbed oxygen, respectively. The main signal with the binding energy at 284.58 eV in the C 1s spectrum (Figure 4b) corresponds to the C‐C bond with sp^2^ hybridization.^[^ ^19^, ^20^, ^21^ ^]^ The other two small signals observed at 285.41 eV and 288.06 eV are related to sp^2^ and sp^3^ hybridized C‐N bonding, respectively, that they originate from the substitution of the N atoms, defects or the edge of the graphene sheets.^[^ ^19^, ^20^, ^21^ ^]^ The N 1s XPS spectrum (Figure 4c) can be deconvoluted to three individual peaks characteristic to pyridinic N (398.12 eV), pyrrolic N (399.39 eV) and graphitic N (400.25 eV).^[^ ^22^, ^23^ ^]^ From Figure 4b,c, the N atom to C atom ratio and the percentages of the graphitic N, pyridinic N and pyrrolic N are calculated and tabulated as in **Table** **1**.

**Table 1:** The N atom to C atom ratio and the percentages of the graphitic N, pyridinic N and pyrrolic N (Data were calculated from Figure 4b and Figure 4c).

Graphene Film	$\frac{N\;\mathrm{Atoms}}{C\;\mathrm{Atoms}}$	Graphitic N [atom%]	Pyrrolic N [atom%]	Pyridinic N [atom%]
U30	1.34	54.01	42.20	3.79

**Figure 5:**
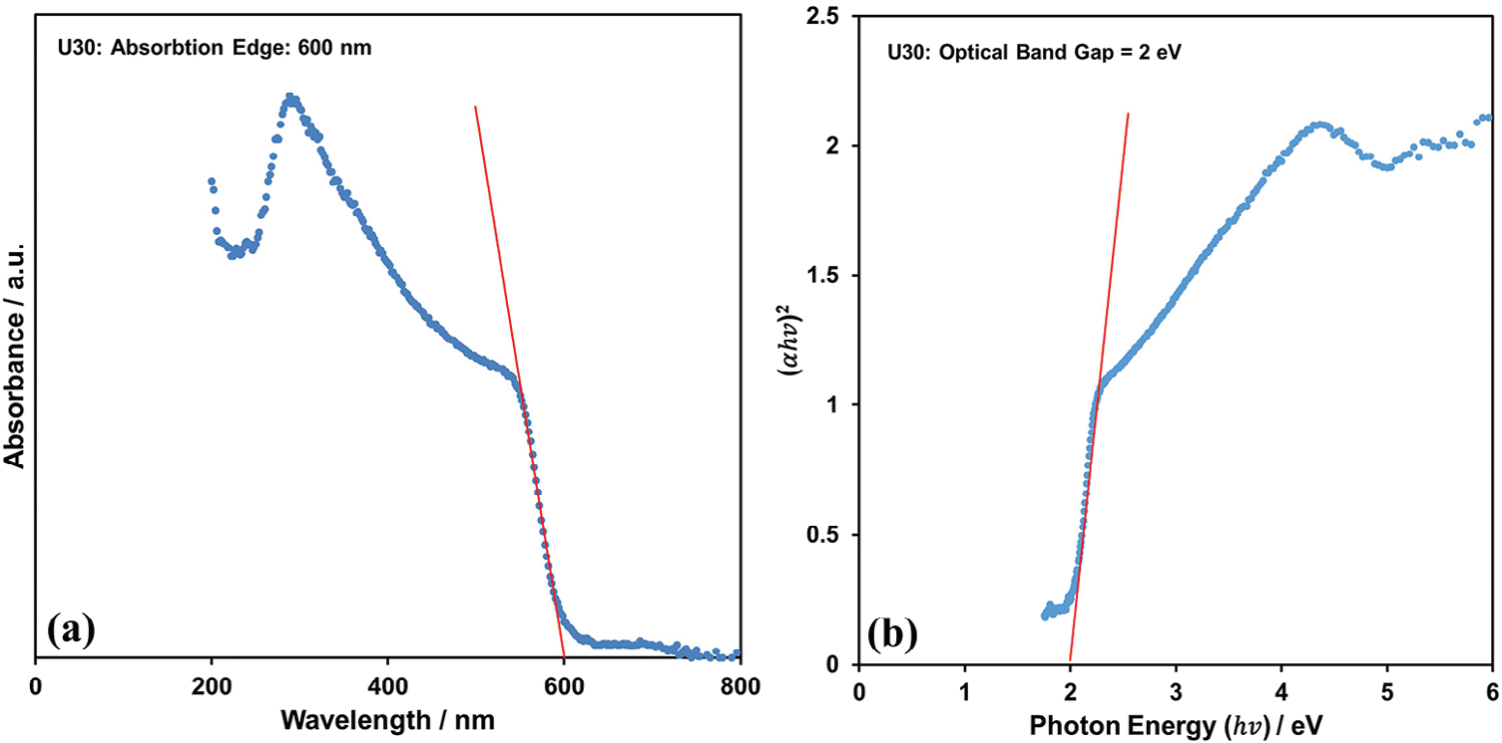
a) The absorption edge and b) optical band gap of U30 graphene film.

The UV‐Vis diffuse reflectance spectra of the U30 graphene film provides the absorption band edge as 600 nm in **Figure** **5**a. The corresponding optical band gap (Eg) can be obtained by Tauc plot^[^ ^16^, ^24^ ^]^ from the data in Figure 5a as in Figure 5b that it is ≈2.0 eV.

Since the photo‐assisted charging with the aid of *I*_3_^−^/*I* ^−^ redox shuttling is only possible if *I* ^−^ ions are oxidized to *I*_3_^−^ ions by the photo‐excited holes of the photo‐catalyst, the valance band (VB) edge potential of the photo‐catalyst must be greater than the redox potential of Reaction 1:^[^ ^1^, ^2^ ^]^

(1) $$I_{3}^{-}+2e^{-}↔3I^{-}\;E=3.586\;V_{{\mathrm{Li}}^{+}/\mathrm{Li}}$$

**Figure 6:**
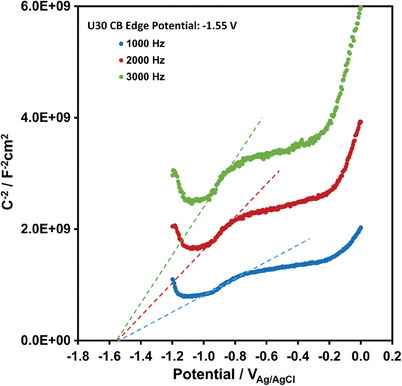
Mott‐Schottky plots of U30 obtained at 1000 Hz, 2000 Hz, and 3000 Hz. The data were gathered after transferring U30 to ITO‐coated glass surface.

The VB edge potential can be calculated by adding up the optical band gap (Eg) and the CB edge potential.^[^ ^16^ ^]^ If the flat band potential is assumed approximately equal to the CB edge potential, Mott‐Sckottky plots, as in **Figure** **6**, may provide CB edge potential. U30 graphene film looks to have a CB edge potential of ≈−1.55 V_Ag/AgCl_, as in Figure 6. The Eg, CB and calculated VB edge potentials are provided in **Table** **2**.

**Table 2:** Eg, CB, and VB values of N‐doped graphene film (U30).

Photo‐catalyst	CB Edge Potential		Optical Band Gap [eV]	VB Edge Potential [V_Li+/Li_]
	V_Ag/AgCl_	V_Li+/Li_		
U30	−1.55	1.71	2.00	3.71

### 3.2 Photo‐Assisted Charging of Li‐Ion Oxygen Battery

Lithium peroxide (Li_2_O_2_) is the main discharge product as in Reaction 2 in the Li‐ion oxygen batteries. During the charging, however, the decomposition of Li_2_O_2_, which has the insulating characteristics, requires a very high overpotential (well above 2.96 V_Li+/Li_).^[^ ^16^ ^]^ It is reported that the redox shuttling (I^−^/I_3_^−^ redox couple: Reaction 1) between the oxygen electrode and Li_2_O_2_ particles assists the decomposition of Li_2_O_2_.^[^ ^1^, ^2^ ^]^ Upon exposure to the visible light, I^−^ ions are oxidized to I_3_^−^ ions by the photo‐excited holes of the photo‐catalyst as in Reaction 3. Subsequently, I_3_^−^ ions diffuse to the cathode surface to reduce back to I^−^ ions as in Reaction 1 which spontaneously drives oxidation of Li_2_O_2_ to Li^+^ ions and O_2_. Meanwhile, the photo‐excited electrons of the n‐type photo‐catalyst flow to the anode to reduce Li^+^ ions as schematically displayed in the graphical abstract. The overall result is that the charge potential of the Li‐ion oxygen battery is reduced significantly upon compensation by the generated photo‐voltage.^[^ ^1^, ^2^ ^]^ The tabulated values in Table 2 are also provided in Figure S3 (Supporting Information) that the synthesized N‐doped graphene film in this work is convenient photo‐catalyst to drive the photo‐assisted charging mechanism for the Li‐ion oxygen battery.

(2) $$2{\mathrm{Li}}^{+}+O_{2}+2e^{-}↔{\mathrm{Li}}_{2}O_{2}\;E=2.96\;V_{{\mathrm{Li}}^{+}/\mathrm{Li}}$$

(3) $$3I^{-}+2h^{+}\to I_{3}^{-}$$

In our previous work, we synthesized nanocomposite photo‐catalysts (g‐C_3_N_4_/rGO) and we loaded them onto the gas diffusion layer to make the photo‐electrode for the photo‐assisted charging of the Li‐ion oxygen battery.^[^ ^16^ ^]^ It was presented clearly by the constant capacity (at 2500 mA h g^−1^ (0.25 mA h cm^−2^)) discharge/charge tests (up to 50 cycles) that it is possible to get very stable discharge capacities at high current densities (up to 300 mA g^−1^ (3 × 10^−2^ mA cm^−2^)) by improving the visible light harvesting performances of the photo‐catalysts.^[^ ^16^ ^]^ In the present work N‐doped graphene (U30) is synthesized on one side of the Cu foam by CVD as the photo‐catalyst and the Cu foam is directly mounted to the homemade cell after porous Pd@rGO cathode loading to the other side of the foam (for simplicity whole transfer‐free process will be referred by “direct synthesis” in the rest of the paper) for the photo‐assisted charging of the Li‐ion oxygen battery.

**Figure 7:**
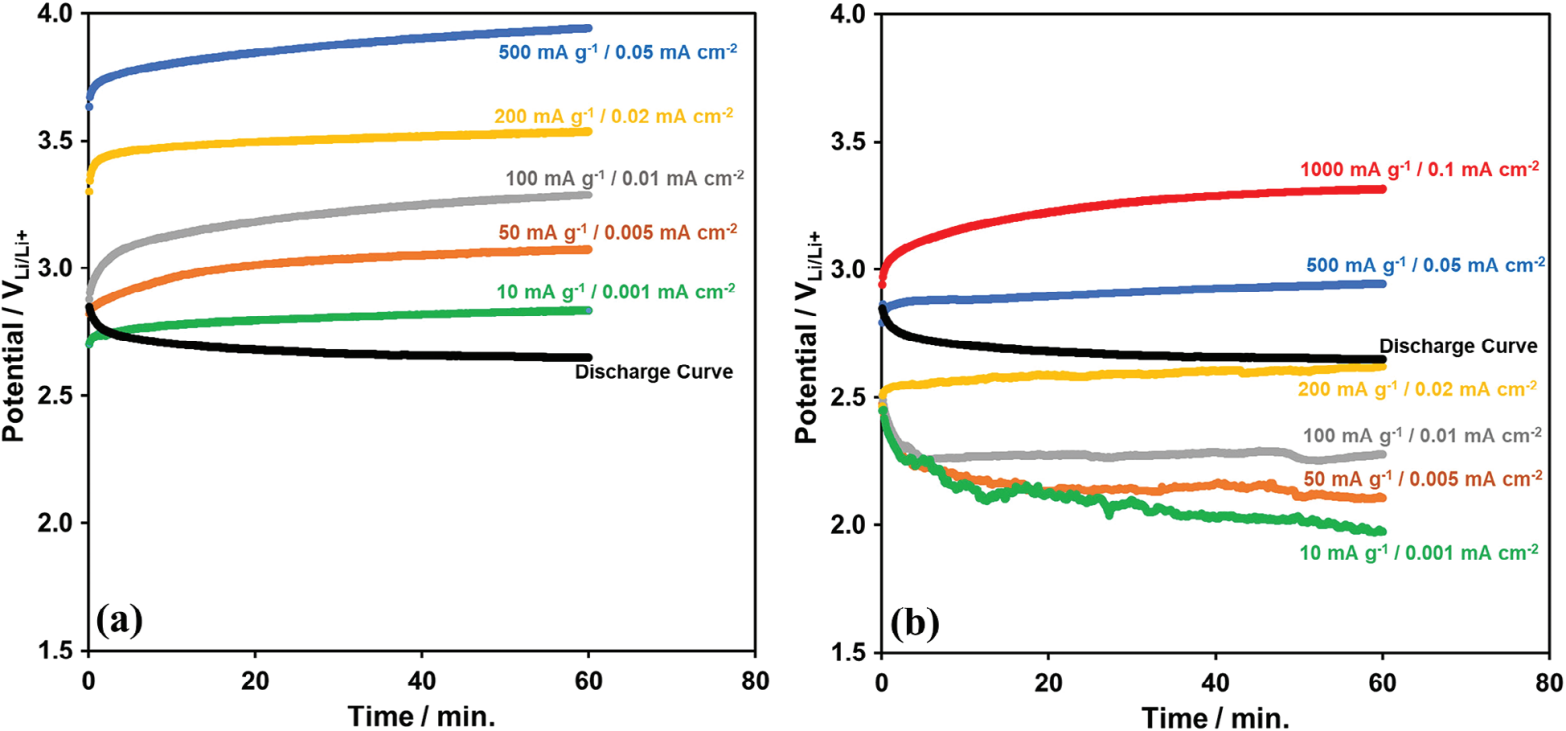
1 h–long charge curves, at various current densities, of Li‐ion oxygen battery under a) dark and b) photo‐assisted (with the directly synthesized transfer free N‐doped graphene photo‐catalyst) conditions. The discharge curve, for comparison, is also provided.

The 1 h‐long charge curves (at various current densities) of Li‐ion oxygen battery with porous Pd@rGO cathode, and the electrolyte without LiI under the dark conditions are provided in **Figure** **7**a. As expected, as the charge current densities increase, the charge potential approaches 4.0 V_Li+/Li_ even for a short period. For comparison, the 1 h‐long charge curves are obtained with the directly synthesized N‐doped graphene (U30) photo‐catalyst under the photo‐assisted charging condition with the electrolyte including LiI as in Figure 7b. The charge potential decreases down to 2 V_Li+/Li_ at 10 mA g^−1^ (0.001 mA cm^−2^), and it remains below the discharge potential (2.65 V_Li+/Li_) up to the 200 mA g^−1^ (0.02 mA cm^−2^) charging current density in Figure 7b. After 1 h photo‐charging, the charge potentials are 2.65 V_Li+/Li_ and 3.32 V_Li+/Li_ at 500 mA g^−1^ (0.05 mA cm^−2^) and 1000 mA g^−1^ (0.1 mA cm^−2^) current densities, respectively, in Figure 7b. These high‐current‐density (0.05 mA cm^−2^ and 0.1 mA cm^−2^) charge potentials observed in Figure 7b are lower than those reported in the literature.^[^ ^1^, ^2^, ^12^, ^13^, ^14^, ^15^, ^16^ ^]^ Obviously, the few atomic layer photo‐catalyst synthesized directly on the current collector acts as very efficient photo‐electrode by minimizing the degradations resulting from the electron‐hole recombination.

**Figure 8:**
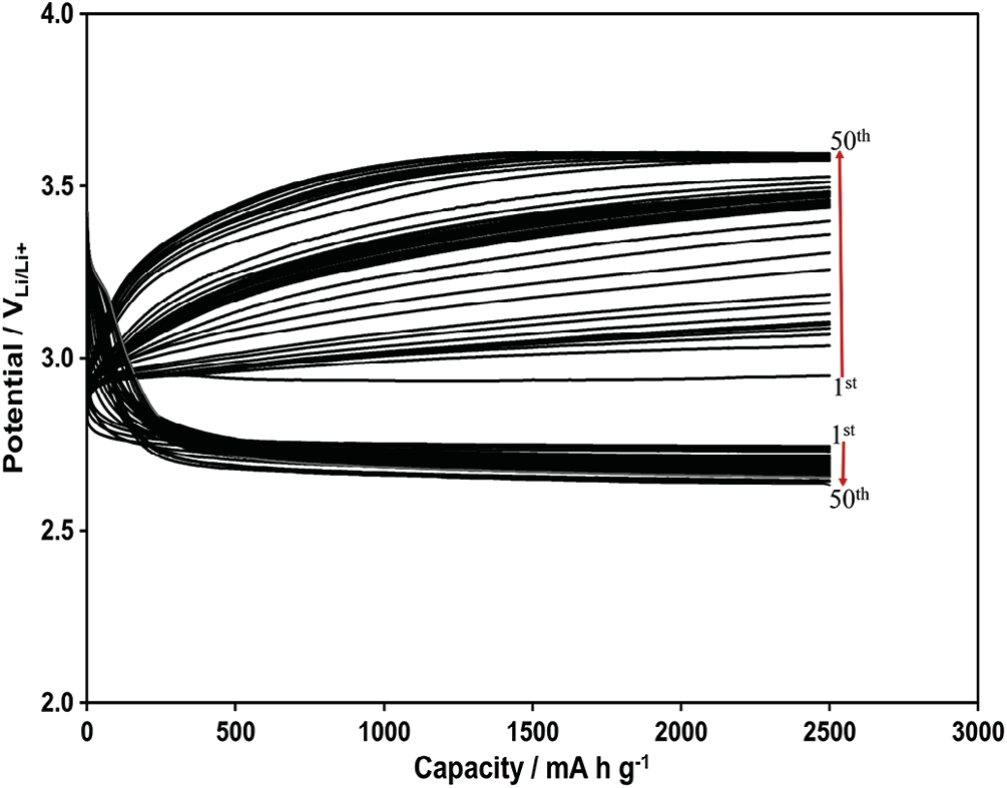
The fifty cycle discharge and charge curves gathered at 2500 mA h g^−1^ (0.25 mA h cm^−2^) constant capacity and 1000 mA g^−1^ (0.1 mA cm^−2^) current density for Li‐ion oxygen battery with the directly synthesized transfer free N‐doped graphene photo‐catalyst, porous Pd@rGO cathode and electrolyte including LiI under the photo‐assisted condition.

The efficiency of the directly synthesized N‐doped graphene photo‐catalyst is also evaluated by the discharge/charge cyclic test of the Li‐ion oxygen battery. Discharge and charge curves gathered at 2500 mA h g^−1^ (0.25 mA h cm^−2^) constant capacity and 1000 mA g^−1^ (0.1 mA cm^−2^) current density for the fifty cycles under the photo‐assisted condition are shown in **Figure** **8**. In order to show clearly the effectiveness of the photo‐assistance, the 1st, 10th, 30th and 50th charge and discharge curves of Figure 8 are compared with those obtained under the dark (in the absence of the photo‐assistance) condition in Figure S4 (Supporting Information). Obviously, the photo‐assistance improves the cyclic performance of the Li‐ion oxygen battery significantly. Previously we obtained 2500 mA h g^−1^ (0.25 mA h cm^−2^) constant discharge capacity without degradation for 50 cycles with g‐C_3_N_4_ and g‐C_3_N_4_/rGO nanocomposite photo‐catalysts up to 200 mA g^−1^ (0.02 mA cm^−2^) and 300 mA g^−1^ (0.03 mA cm^−2^) current densities, respectively.^[^ ^16^ ^]^ The directly synthesized N‐doped graphene photo‐catalyst, however, extends the applied current density up to 1000 mA g^−1^ (0.1 mA cm^−2^) without causing degradation for 50 cycles in the discharge capacity in Figure 8 that this value has very critical importance for the commercial applications.

The photo‐assistance not only reduces the charge potential and improves the charging behavior but also causes discharge potential to increase as in Figure 8 with the same photo‐assistance mechanism. This effect can be observed more clearly by comparing the curves in Figure S4a and S4b (Supporting Information). Therefore, the contribution of the photo‐assistance to the performance of the Li‐ion oxygen battery can be expressed by the charge and discharge overpotentials. The charge/discharge cycle dependencies of both the charge and discharge overpotentials are provided at the photo‐assisted and dark conditions in Figure S5 (Supporting Information). The charge curves reach to the maximum charging overpotential (3.6 V_Li/Li+_ (charge cut‐off potential)–2.8 V_Li/Li+_ (open circuit potential) = 0.8) at the 50th cycle and 21th cycle at the photo‐assisted and dark conditions, respectively. While the discharge curves at the dark condition reach again to the maximum overpotential (2.8 V_Li/Li+_–2.0 V_Li/Li+_ (discharge cut‐off potential) = 0.8) at the 21th cycle, those in the photo‐assisted condition have only 0.17 V overpotential at the 50th cycle. The overall conclusion is that the photo‐assistance improves both the charging and discharging process of the Li‐ion oxygen battery.

Yu et al.,^[^ ^1^ ^]^ underlined the theoretical basis for selecting the convenient photo‐catalyst for the photo‐assisted charging of the Li‐ion oxygen battery. Theoretical convenience, however, does not guarantee the practical efficiency since the photo‐catalysts suffer the degradations arising from the electron‐hole recombination especially if the thickness is not homogeneous or the complications arising from the loading process of the photo‐catalysts to current collectors. This work presented clearly that the direct synthesis of the few atomic layer 2D photo‐catalyst on the current collector without needing any external loading or transferring processes is a very effective pathway in the improvement of the photo‐assisted charging performance of the Li‐ion oxygen battery.

## 4 Conclusion

In conclusion, N‐doped graphene film photo‐catalyst was synthesized by CVD aiming the effective photo‐assisted charging of the Li‐ion oxygen battery. Optical characterizations showed that the n‐type graphene film had a 2.00 eV optical band gap and 3.71 V_Li+/Li_ VB edge potential. XPS spectra provided that 1.34 atomic% of the graphene film was doped N atoms and 54.01 atomic% of the doped N atoms were graphitic N (the rest of the N atoms were pyridinic and pyrrolic). The photo‐assisted charging tests indicated that the N‐doped graphene film photo‐catalyst reduced the charging potential significantly even at 1000 mA g^−1^ (0.1 mA cm^−2^) current density. This work showed clearly that the directly synthesized 2D photo‐catalyst on the current collector without needing any external loading or transferring processes provided a striking light‐harvesting performance. Thus, not only the structural characteristics of the photo‐catalyst but also the photo‐electrode preparation approach contributes significantly to the efficient generation of the photo‐voltage for the photo‐assisted charging of the Li‐ion oxygen battery.

## Conflict of Interest

The authors declare no conflict of interest.

## Supporting information

Supporting Information

## Data Availability

The data that support the findings of this study are available from the corresponding author upon reasonable request.

## References

[1] M. Yu , X. Ren , L. Ma , Y. Wu , Nat. Commun. 2014, 5, 1. 10.1038/ncomms6111 25277368

[2] Y. Liu , N. Li , S. Wu , K. Liao , K. Zhu , J. Yi , H. Zhou , Energy Environ. Sci. 2015, 8, 2664.

[3] X. Wang , G. Sun , P. Routh , D.‐H. Kim , W. Huang , P. Chen , Chem. Soc. Rev. 2014, 43, 7067. 24954470 10.1039/c4cs00141a

[4] D. Akinwande , C. Huyghebaert , C.‐H. Wang , M. I. Serna , S. Goossens , L.‐J. Li , H.‐S. P. Wong , F. H. L. Koppens , Nature 2019, 573, 507. 31554977 10.1038/s41586-019-1573-9

[5] Y. Xue , B. Wu , Q. Bao , Y. Liu , Small 2014, 10, 2975. 24715648 10.1002/smll.201400706

[6] Y. Shi , K. K. Kim , A. Reina , M. Hofmann , L.‐J. Li , J. Kong , ACS Nano 2010, 4, 2689. 20433163 10.1021/nn1005478

[7] W. J. Yu , Y. Liu , H. Zhou , A. Yin , Z. Li , Y. Huang , X. Duan , Nat. Nanotechnol. 2013, 8, 952. 24162001 10.1038/nnano.2013.219 PMC4249654

[8] S. Bae , H. Kim , Y. Lee , X. Xu , J.‐S. Park , Y. Zheng , J. Balakrishnan , T. Lei , H. Ri Kim , Y. I. Song , Y.‐J. Kim , K. S. Kim , B. Özyilmaz , J.‐H. Ahn , B. H. Hong , S. Iijima , Nat. Nanotechnol. 2010, 5, 574. 20562870 10.1038/nnano.2010.132

[9] R. Zan , A. Altuntepe , J. Mol. Struct. 2020, 1199, 127026.

[10] G. Deokar , J. Jin , U. Schwingenschlögl , P. M. F. J. Costa , npj 2D Mater. Appl. 2022, 6, 1.

[11] D. Wei , Y. Liu , Y. Wang , H. Zhang , L. Huang , G. Yu , Nano Lett. 2009, 9, 1752. 19326921 10.1021/nl803279t

[12] X.‐Y. Yang , X.‐L. Feng , X. Jin , M.‐Z. Shao , B.‐L. Yan , J.‐M. Yan , Y. Zhang , X.‐B. Zhang , Angew. Chemie 2019, 131, 16563.

[13] S. Tong , C. Luo , J. Li , Z. Mei , M. Wu , A. P. O'mullane , H. Zhu , Angew. Chemie 2020, 132, 21095. 10.1002/anie.202007906 32761724

[14] M. Li , X. Wang , F. Li , L. Zheng , J. Xu , J. Yu , Adv. Mater. 2020, 32, 1. 10.1002/adma.201907098 32671896

[15] H. Xue , T. Wang , Y. Feng , H. Gong , X. Fan , B. Gao , Y. Kong , C. Jiang , S. Zhang , X. Huang , J. He , Nanoscale 2020, 12, 18742. 32970089 10.1039/d0nr04956e

[16] E. Lök , N. Kaçar , M. Çayirli , R. C. Özden , M. Anik , ACS Appl. Mater. Interfaces 2022, 14, 34583. 35861585 10.1021/acsami.2c05607 PMC9354003

[17] A. C. Ferrari , Solid State Commun. 2007, 143, 47.

[18] A. C. Ferrari , J. C. Meyer , V. Scardaci , C. Casiraghi , M. Lazzeri , F. Mauri , S. Piscanec , D. Jiang , K. S. Novoselov , S. Roth , A. K. Geim , Phys. Rev. Lett. 2006, 97, 1. 10.1103/PhysRevLett.97.187401 17155573

[19] C. Ronning , H. Feldermann , R. Merk , H. Hofsäss , P. Reinke , J.‐U. Thiele , Phys. Rev. B – Condens. Matter Mater. Phys. 1998, 58, 2207.

[20] J. W. Jang , C. E. Lee , S. C. Lyu , T. J. Lee , C. J. Lee , Appl. Phys. Lett. 2004, 84, 2877.

[21] X. Dong , P. Wang , W. Fang , C.‐Y. Su , Y.‐H. Chen , L.‐J. Li , W. Huang , P. Chen , Carbon N. Y. 2011, 49, 3672.

[22] X. Wang , Y. Liu , D. Zhu , L. Zhang , H. Ma , N. Yao , B. Zhang , J. Phys. Chem. B 2002, 106, 2186.

[23] J. Casanovas , J. M. Ricart , J. Rubio , F. Illas , J. M. Jiménez‐Mateos , J. Am. Chem. Soc. 1996, 118, 8071.

[24] X.‐J. Wang , Q. Wang , F.‐T. Li , W.‐Y. Yang , Y. Zhao , Y.‐J. Hao , S.‐J. Liu , Chem. Eng. J. 2013, 234, 361.

