# Supplementary material for: Synthesis of N‐Doped Graphene Photo‐Catalyst for Photo‐Assisted Charging of Li‐Ion Oxygen Battery
Source: Glob Chall. 2023 Dec 7;8(1):2300166. doi: 10.1002/gch2.202300166 (PMC10784194; doi:10.1002/gch2.202300166)
Supplement: Supplementary file 1 — Supporting Information [file GCH2-8-2300166-s001.pdf]

Global Challenges
Open Access

Supporting Information

for *Global Challenges.*, DOI 10.1002/gch2.202300166

Synthesis of N-Doped Graphene Photo-Catalyst for Photo-Assisted Charging of Li-Ion Oxygen Battery

*Nilay Kaçar, Ersu Lökçü, Meltem Çayirli, Reşat Can Özden, Sahin Coskun, Cigdem Toparli, İbrahim Çelikyürek and Mustafa Anik**

Supporting Information

Synthesis of N-Doped Graphene Photo-Catalyst for Photo-Assisted Charging of Li-Ion Oxygen Battery

Nilay Kaçar[a,¥], Ersu Lökçü[a], Meltem Çayirli[a], Reşat Can Özden[a], Şahin Çoşkun[a], Çiğdem Toparlı[b], İbrahim Çelikyürek[a] and Mustafa Anik[a,*]

[a] Department of Metallurgical and Materials Engineering, Eskisehir Osmangazi University, 26040, Eskisehir, Turkey

[b] Department of Metallurgical and Materials Engineering, Middle East Technical University, 06800, Ankara, Turkey

[¥] Current Address: Department of Metallurgical and Materials Engineering, Bursa Technical University, 16310, Bursa, Turkey

*Corresponding author: manik@ogu.edu.tr

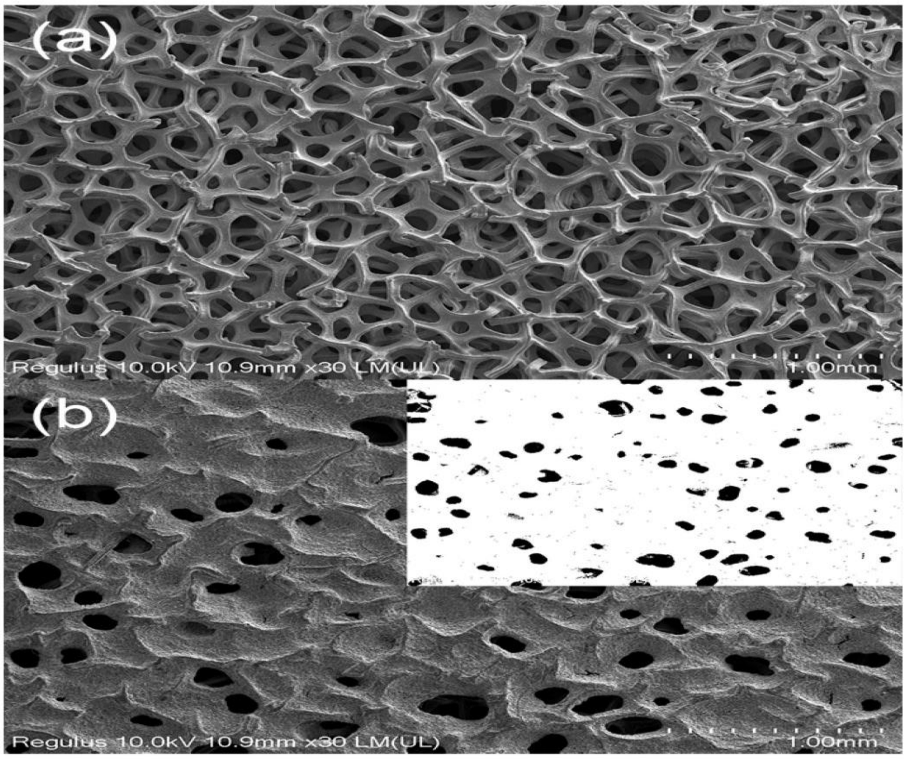

Figure S1. SEM images of the (a) pristine copper foam (550 g/m^2) and (b) surface after loading of the cathode active material. The threshold image inset provides that the cathode loaded surface is approximately 91% of the geometrical area.

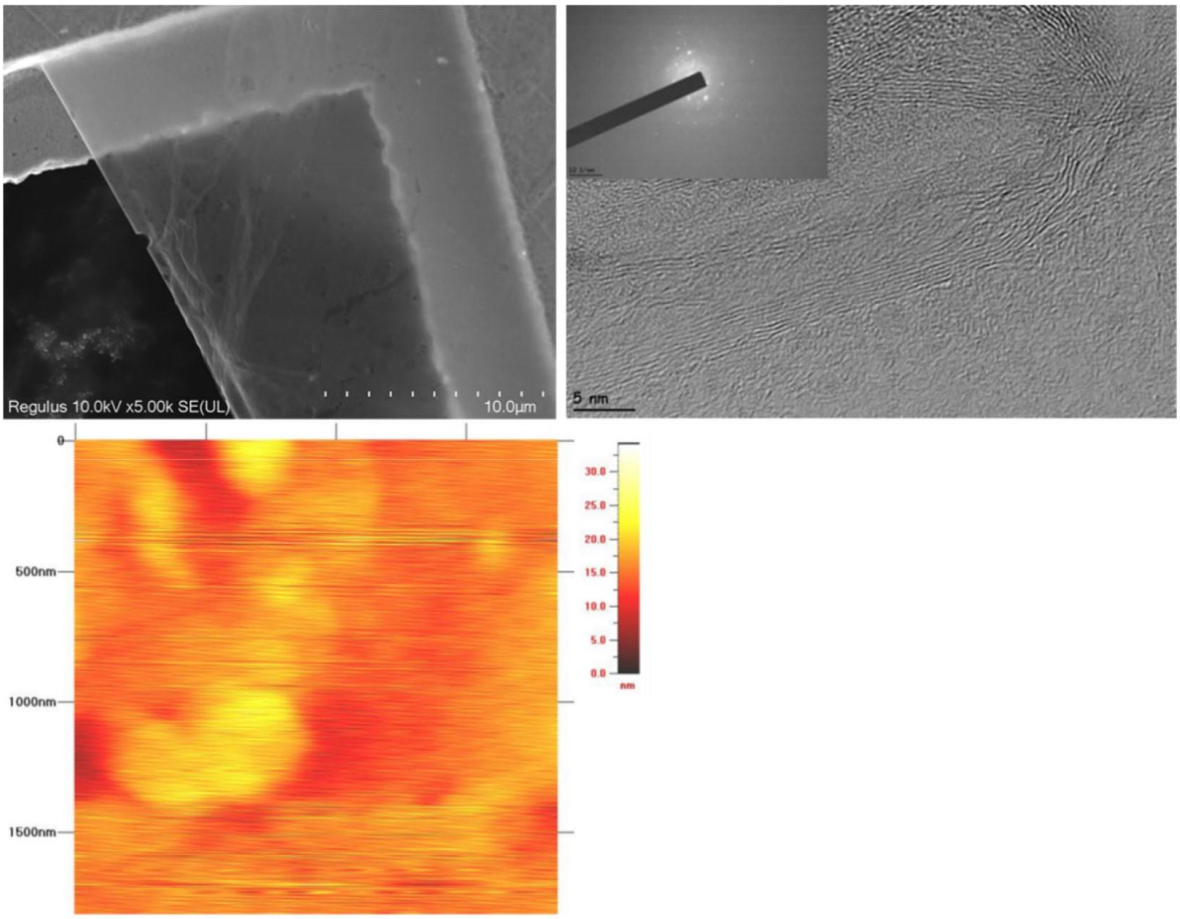

Figure S2. The (a) SEM, (b) TEM and (c) AFM images of the synthesized U30 films.

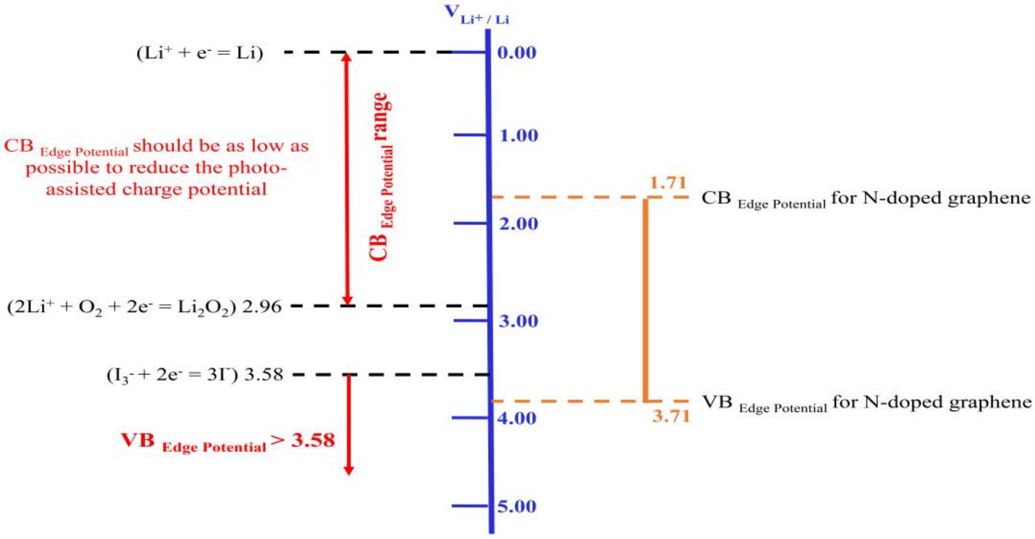

Figure S3. Positions of the CB and VB edge potentials of the N-doped graphene in the visible light utilization scale for the photo-assisted charging of the Li-ion oxygen battery.

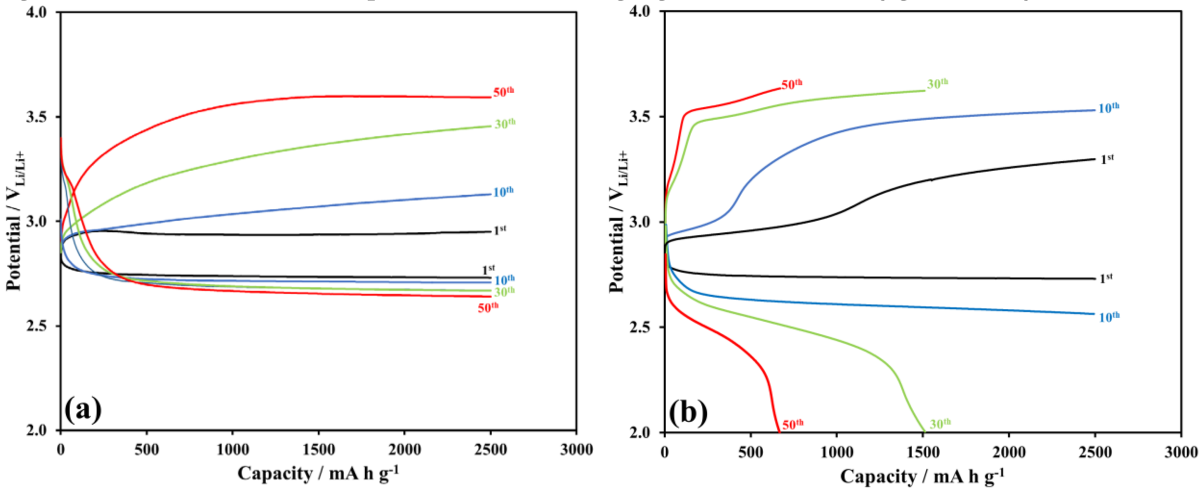

Figure S4. 1st, 10th, 30th and 50th charge and discharge curves of Li-ion oxygen battery at (a) photo-assisted and (b) dark conditions.

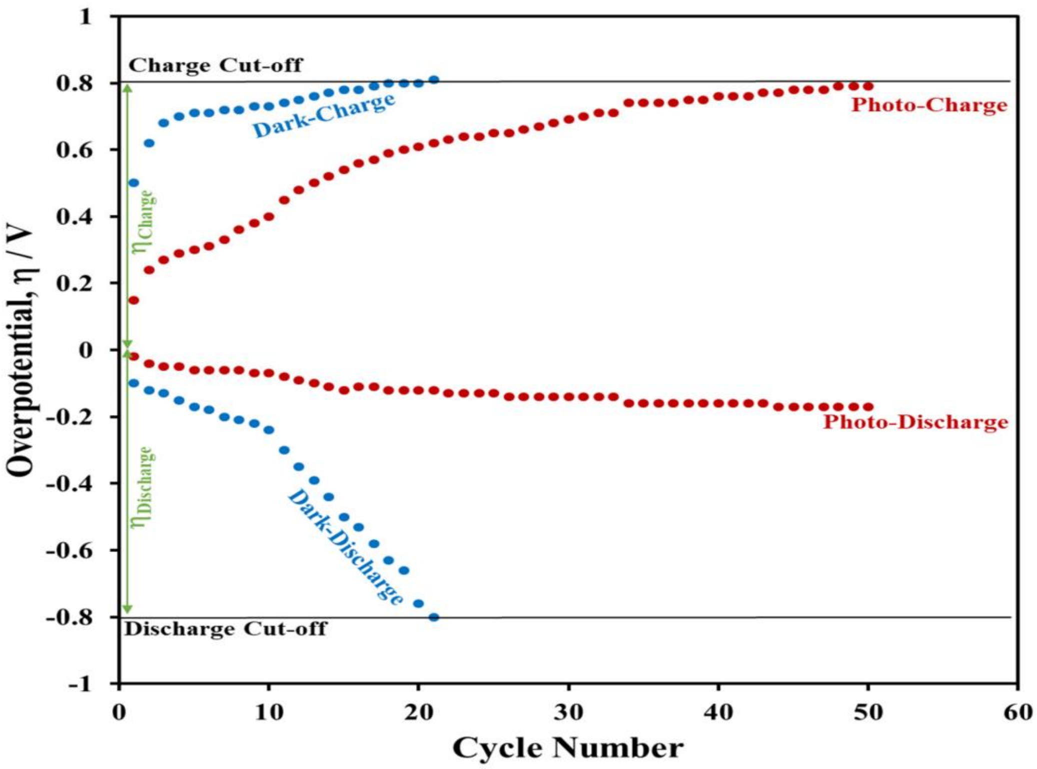

Figure S5. Charge/discharge cycle dependence of the overpotentials at both the photo-assisted and dark conditions.
